# Non-Invasive radial pressure wave analysis may digitally predict women’s risks of type 2 diabetes (T2DM): A case and control group study

**DOI:** 10.1371/journal.pone.0259269

**Published:** 2021-10-29

**Authors:** Chih-Yu Chen, Kuo-meng Liao, Sheng-Hung Wang, Su-Chiu Chen, Chen-Jung Chang, Tien-Chung Wang, Gin-Chung Wang

**Affiliations:** 1 Department of Obstetrics and Gynecology, Keelung Hospital of the Ministry of Health and Welfare, Keelung, Taiwan; 2 Department of Health Care Management, National Taipei University of Nursing and Health Sciences, Taipei, Taiwan; 3 Institute of Clinical Medicine, National Yang-Ming University, Taipei, Taiwan; 4 Division of Endocrinology & Metabolism of Zhongxiao Branch of Taipei City Hospital, Taipei, Taiwan; 5 MiiAnn Medical Research Center, Taipei, Taiwan; 6 College of Public Health, National Taiwan University, Taipei, Taiwan; 7 The Department of Food and Nutrition from Seoul National University, Seoul, South Korea; 8 JinMu Health Technology, Taipei, Taiwan; Indiana University Purdue University at Indianapolis, UNITED STATES

## Abstract

**Background:**

Women not only have worse diabetes complications, but also have menstrual cycle, pregnancy, and menopause which can make managing diabetes more difficult. The aim of this study was to investigate if radial pressure wave analysis may non-invasively screen for women’s risk of type 2 diabetes.

**Methods:**

Spectrum analysis of the radial pressure wave was performed to evaluate the first five harmonic components, C1 to C5. The study consisted of a total of 808 non-pregnant female subjects aged 20–95 over the period of 4 years, and 404 of them were diagnosed with Type 2 diabetes as the case group.

**Result:**

The first five harmonic components are significantly different in a comparison of the case group and the control group. In the logistic regression analysis, T2DM was found to be associated with C1 (OR = 1.055, CI = 1.037–1.074, p < 0.001), C2 (OR = 1.051, CI = 1.019–1.085, p = 0.002), and C3 (OR = 0.972, CI = 0.950–0.994, p = 0.013). In the Receiver Operating Characteristic curve analysis, the Area Under Curve of using C3 only (70%, p <0.05), weighted C1, C2 and C3, (75%, p < 0.05), and weighted C1, C2 and C3 and Body mass Index (84%, p <0.05) were tested for the accuracy on how well these tests separate the women into the groups with and without the T2DM.

**Conclusion:**

We thus concluded that pulse spectrum was a non-invasive predictor for women’s risk of T2DM.

## 1. Introduction

Pregnancy and menopause have profound effects on the risks for diabetes and therefore warrant more aggressive diagnostic attention and monitoring [1]. Women who have diabetes are twice as likely to develop heart disease compared to men [2]. The difference between all-cause mortality rates in women with T2DM and those without T2DM has more than doubled [3]. Wearable and non-invasive risk assessments make the large-scale screening for diabetes feasible. In addition to the widely used risk factors such as age and body mass Index (BMI) [4], incorporating non-invasive measurement strategies may further advance the prevention of diabetes for women in a cost effective and convenient way [5].

Radial pressure wave describes the change of the arterial pressure over time and provides information of the condition of ventricular-arterial system [6]. Changes in blood flow and the functioning of organs will alter the waveform of the radial pulse wave [7]. The 12-second non-invasive screening may especially provide an alternative for those women in remote regions and lower-middle-income countries who are less likely to be tested and diagnosed for their diabetes [8].

In our previous study, the harmonic indexes of radial pulse could be risk factors for complications of diabetes especially for the cardiovascular complications [9]. Beside, we found that the changes in female radial wave harmonics occur in the menstrual cycle [10], pregnancy [11], menopause [12], and dysmenorrhea [13]. Harmonic index have been shown to be associated with diabetic complications and some physiological changes in women, in this study we will further investigate the association between harmonic index and diabetes in women. Therefore, the aim of this study was to investigate if radial pressure wave analysis may non-invasively screen for women’s risk of type 2 diabetes.

## 2. Methods

### 2.1 Study population

The subjects of this study were all Asians, aged 20–95 years, recruited by Taipei City Hospital between August 2016 and February 2020. Both oral and written information about the study was given to the patients. Informed consent was obtained from all patients after receiving approval from the Institutional Review Board of Taipei City Hospital (IRB number: ISRCTN20480882). The case group of women with type 2 diabetes were under the care of the Division of Endocrinology & Metabolism at Zhongxiao Branch and the control group were patients randomly selected from the Department of Gynecology and Obstetrics at the Renai Branch. Patients with diabetes, major cardiovascular disease, kidney disease, liver disease and cancer would seriously affect the circulatory system were excluded from the control group, and pregnant subjects were excluded from this case and control analysis.

### 2.2 Study design

A non-invasive radial pulse measurement was employed to test whether there is a relationship between type 2 diabetes (T2DM) and the first five harmonics of the radial pulse. Each subject’s radial pressure wave data was continuously recorded for 12-seconds using a medical grade pulse measuring instrument (TD01C, MIIANN, Taiwan). Briefly, a piezoresistive sensor is used to record pressure waveforms from the radial artery of the wrist. TD01C proved its intrinsic reliability using artificial pulse generator [14]. The intra-observer and inter-observer reliability of TD01C has also been demonstrated in the previous clinical study [15]. Radial pulse waves measured the pressure in the arteries over time and converted the pressure wave of the radial pulse into frequency waves. Spectrum analysis of the radial pressure wave was then performed to evaluate the first five harmonic components Cn (n = 1–5). Cn is defined by the following equation:

Cn=An/A0 (1)

where A0 is the mean value of pulse wave, and An (n = 1–5) is the nth amplitude coefficient of Fourier series of the radial pulse wave. After radial artery pulse spectrum assessment, upper arm blood pressure was measured with an automated sphygmomanometer (EASY X 800R, JAWON Medical, Korea) according to the instructions. The assessment was performed by trained operators without the presence of physicians and nurses to avoid the white coat effect.

Aging is a known risk factor for T2DM and the risk increases by age [16]. Age also changes specific harmonic components of radial pressure wave [17]. The women diagnosed with T2DM was heavily skewed towards the older age brackets. To eliminate the confounding effect of age in T2DM, we randomly selected and matched case and control subjects by age. If there were no subjects of exactly the same age, the closest age was selected. This process reduced the sample size from 2,106 to 808.

### 2.3 Statistical analysis

All statistical analyses were performed using R version 3.0.2. A T-test was used to compare variables between case and control groups. The logistic regression analysis was used to determine the associations between T2DM and harmonics indexes and the odds ratios. Age, BMI, Systolic blood pressure (SBP) and Diastolic blood pressure (DBP) were controlled by acting as the covariates in the logistic regression model. The Area Under Curve (AUC) was tested for the accuracy on how well these tests separate the women into the groups with and without the T2DM. ROC analysis was used to calculate the sensitivity and specificity of the variables and the cut off points [18]. Lastly, p-value smaller than 0.05 was accepted as statistically significant.

## 3. Results

### 3.1 T-test of case and control groups

In Table 1, a T-test was used to understand the characteristics of the case and control group. BMI and blood pressures were known risk factors for prediction of T2DM. In the in the diabetic group, BMI and SBP were significantly higher. Radial pulse wave harmonic indexes for those patients with T2DM, C1, C2 and C4 were significantly higher, and C3 and C5 were significantly lower.

**Table 1:** T-test for characteristics of the case and control populations.

	Control: Female without T2DM (N = 404)	Case: Female with T2DM (N = 404)	*P*
Age (year)	52.43 ± 13.15	51.76 ± 12.96	0.465
BMI (kg/m^2^)	22.71 ± 3.6	27.44 ± 5.59	<0.001*
SBP (mmHg)	120.95 ± 18.11	125.96 ± 10.96	<0.001*
DBP (mmHg)	72.19 ± 10.76	73.91 ± 8.02	0.018
C1	90.24 ± 11.89	98.21 ± 20.39	<0.001*
C2	48.96 ± 5.42	52.78 ± 9.32	<0.001*
C3	52.72 ± 12.07	44.56 ± 11.39	<0.001*
C4	56.26 ± 22.18	67.01 ± 29.87	<0.001*
C5	105.62 ± 33.78	95.85 ± 38.71	<0.001*

BMI = Body mass Index, SBP = Systolic blood pressure, DBP = Diastolic blood pressure, C1-C5 = 1^st^ to 5^th^ Harmonics Indexes. Asterisks indicate that the means of variables in with type 2 diabetes group differ significantly from without type 2 diabetes group. (p < = 0.001).

### 3.2 Logistic regression

To investigate the association between T2DM and the radial pressure wave harmonics, we apply the logistic regression of the generalized linear model. BMI was controlled by acting as the covariates in the logistic regression model.

After controlling age, BMI, SBP and DBP, C1 and C2 were positively and C3 was negatively associated with T2DM. Odds ratios obtained from logistic regression describe associations of BMI, C1, C2 and C3 with T2DM In logistic regression analysis, T2DM was found to be positively associated with BMI (OR = 1.284, CI = 1.221–1.357, p < 0.001), C1 (OR = 1.055, CI = 1.037–1.074, p < 0.001), C2 (OR = 1.051, CI = 1.019–1.085, p = 0.002), and negatively associated C3 (OR = 0.972, CI = 0.950–0.994, p = 0.013) values in Table 2.

**Table 2:** Result of logistic regression model: Age, BMI, C1, C2 and C3 for type 2 diabetes (N = 808, age 20–95).

	β estimate	Standard Error (SE)	*P-value*	Odds Ratio (OR)	Confidence Interval of OR
(Intercept)	-10.960	2.181	<0.001		
Age (year)	-0.018	0.009	0.045	0.982	0.965–0.999
BMI (kg/m^2^)	0.250	0.027	<0.001	1.284	1.221–1.357
SBP (mmHg)	0.006	0.010	0.560	1.006	0.987–1.027
DBP (mmHg)	-0.006	0.015	0.700	0.994	0.965–1.023
C1	0.053	0.009	<0.001	1.055	1.037–1.074
C2	0.050	0.016	0.002	1.051	1.019–1.085
C3	-0.029	0.012	0.013	0.972	0.950–0.994
C4	0.004	0.006	0.490	1.004	0.993–1.015
C5	-0.005	0.004	0.135	0.995	0.988–1.002

BMI = Body mass Index, SBP = Systolic blood pressure, DBP = Diastolic blood pressure, C1-C5 = 1^st^ to 5^th^ Harmonics Indexes.

### 3.3 ROC analysis

The receiver operating characteristics (ROC) curve of each predictor was obtained, and the AUC was seen as the evaluation criteria of the performance of prediction.

**Fig 1:**
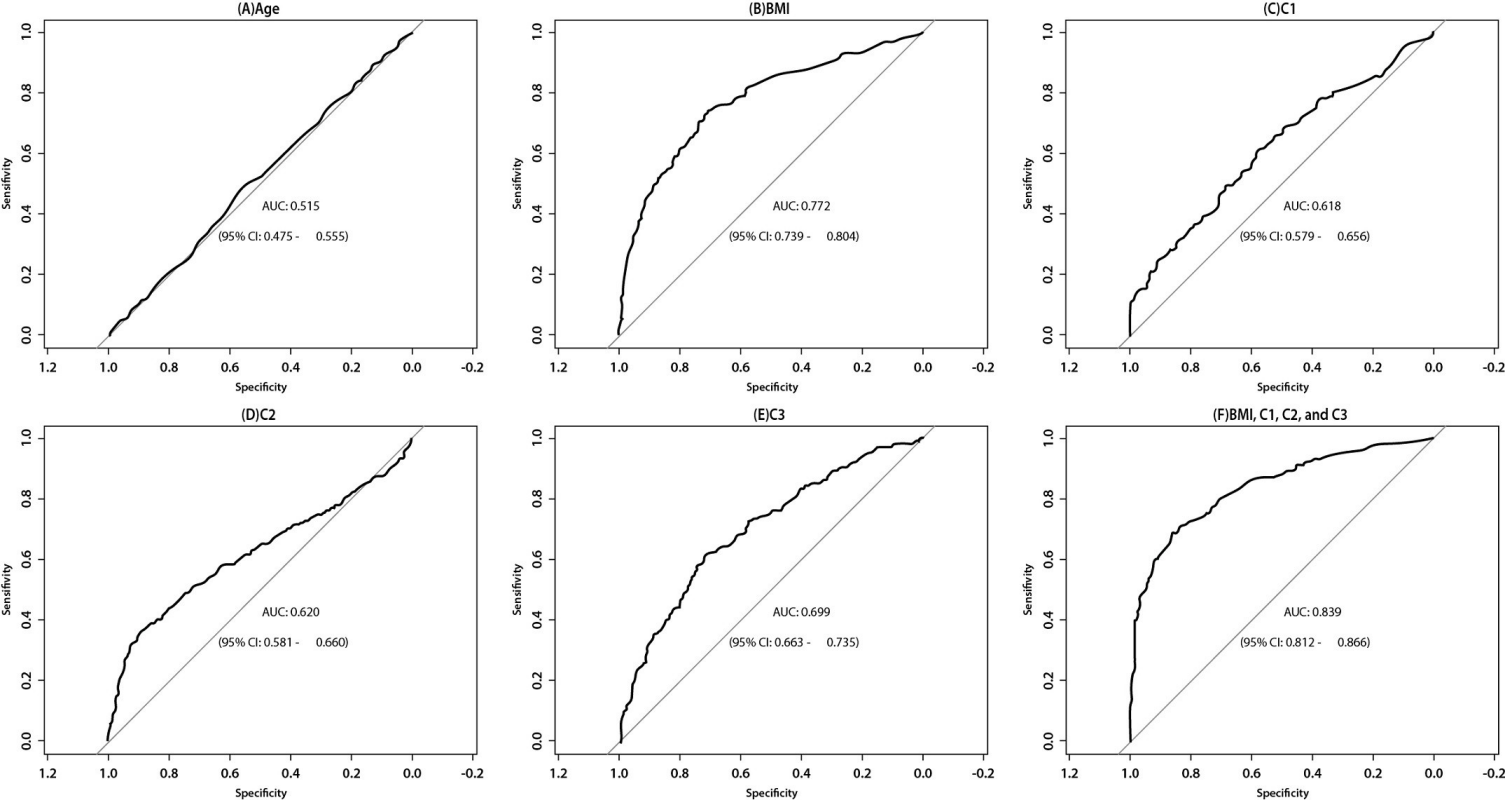
Area Under Curve (AUC) constructed to predict women with T2DM. Comparison of receiver operating characteristic curves for (A) Age, (B) Body mass index (BMI), (C) C1, (D) C2, (E) C3, and (F) BMI and C1, C2 and C3.

Besides the single BMI and C3 predictor, the combination of all the predictors were also tested, with the corresponding coefficients of variables in the logistic regression models were also used as the weighting parameter (Fig 1). Since age as a variable has already been controlled by the previous age-matching procedure, it predicts T2DM with 51.5%. AUC of BMI, C3 and a combination of BMI, C1, 2 and 3 predictions are 77.2%, 69.9% and 83.9%. By incorporating radial pressure wave harmonics, the probability of the correct predictions improved 6.7%.

Since SBP, DBP, C4 and C5 were not significant in the logistic regression model, the cut points are weighted by coefficients of the logistic regression with BMI (β coefficient = 0.247, p < 0.01), C1 (β coefficient = 0.036, p < 0.001), C2 (β coefficient = 0.062, p < 0.001) and C3 (β coefficient = -0.047, p = 0.001) excluding C4 and C5. The cut point with the highest Youden Index is 0.330. At this cut point, the specificity was 0.861, the sensitivity was 0.686.


Index=−10.365+0.247xBMI+0.036xC1+0.062xC2−0.047xC3

We assumed the prevalence of T2DM in female Taiwanese as 10% based on the government study [19]. Based on this assumption, the Negative Predictive Value (NPV) was 0.961 and Positive Predictive Value (PPV) was 0.355. (Table 3)

**Table 3:** Number of subjects of weighted coefficients of BMI, C1, C2 and C3 (Cut point = 0.330, p < 0.001) with and without T2DM.

T2DM	Number of subjects		Total
	Index < = 0.330	Index > 0.330	
Not Diagnosed	348	56	404
Diagnosed	127	277	404
Total	475	333	808

$\text{NPV}=\frac{\text{Specificity}\;\text{x}\;(1-\text{Prevalence})}{\left(1-\text{Sensitivity})\;\text{x}\;\text{Prevalence}+\text{Specificity}\;\text{x}\;(1-\text{Prevalence}\right)}=0.961.$

$\text{PPV}=\frac{\text{Sensitivity}\;\text{x}\;\text{Prevalence}}{\text{Sensitivity}\;\text{x}\;\text{Prevalence}+(1-\text{Specificity})\;\text{x}\;(1-\text{Prevalence})}=0.355.$

**Fig 2:**
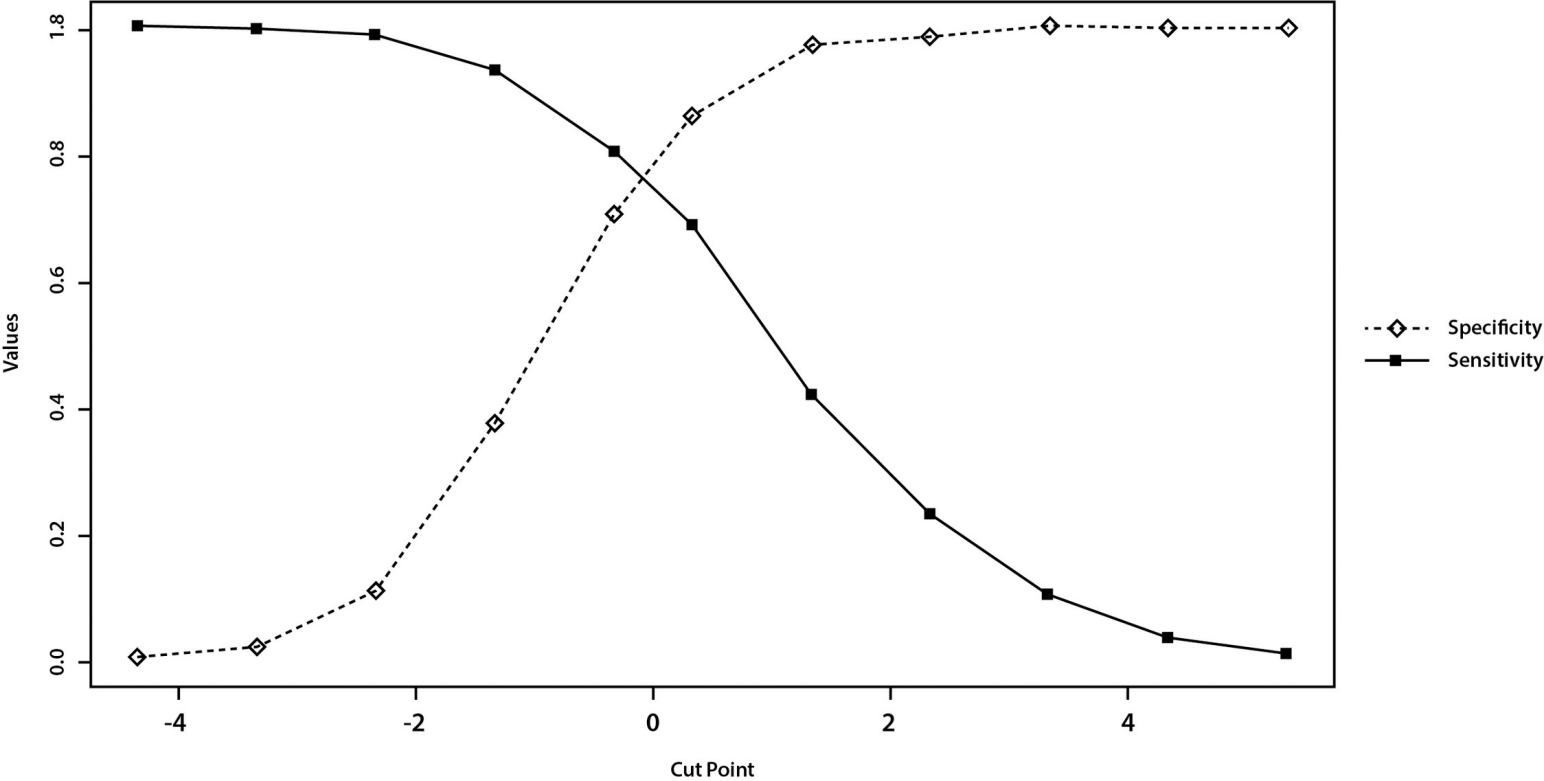
Specificity and sensitivity trade-off at different cut points (weighted coefficients of BMI, C1, C2 and C3).

There is a trade-off between sensitivity and specificity that is dependent on the cut-off level chosen for a positive diagnosis (Fig 2). If those identified by the screening procedure will be assessed in more detail, the assessment will help exclude "false positives" [20]. It may be argued that sensitivity may be more important than specificity for screening T2DM if the follow up diagnostic tests are readily available.

In summary, the study confirmed that general and abdominal obesity are known significant risk factors for diabetes in the future. BMI was strongly associated with diabetes incidence in women [21]. in addition to BMI, C1, C2 and C3 was significantly different between the case and control group of women with or without type 2 diabetes (T2DM). Including radial pulse harmonics as predictors improves AUC of the test by 6.7%.

## 4. Discussion

In a study of 84,000 female nurses for 16 years, it has found that being overweight or obese was the single most important predictor of T2DM [22]. Our study echoed the finding of BMI as the most important predictor. Instead of using BMI as the only predictor, this study provided a non-invasive and low-cost measurement that can increase the accuracy of predicting women with T2DM.

Out of 404 female patients with type 2 diabetes, 24.8% of them were coded with ICD 10 between I00 and I99, diseases of the circulatory system. The relationship between radial pulse waves and status of ventricular-arterial system was illustrated in the past studies [6]. A 1.8 years follow-up demonstrated that higher C1 independently predicts the risk of cardiovascular death, major adverse cardiovascular events, and microvascular outcomes in 2,324 patients with T2DM [9]. Higher C1 may be associated with increased arterial stiffness associated with type 2 diabetes [23].

C2 and C3 were also previously reported to be related to the blood circulation of the renal artery and superior mesenteric artery (SMA) respectively. Young et al. informed that 3 seconds ligating of the left renal artery caused changes to C2 and ligating the SMA caused changes to C3 [24]. 42.9% of our case group were diagnosed with stage one of chronic kidney disease (CKD). The kidney vascular system exhibits a resonant frequency at the second harmonic of the heartbeat [24]. Diabetes is characterized consistently by increases in renal blood flow [25]. Other study reported increased renal vascular resistance of the main renal arteries may imply the presence of any type of underlying renal damage resulting in endothelial dysfunction in type 2 diabetes [26].

Many studies [27] have been based on molecular research to investigate the mechanism of the pancreas to reduce insulin secretion while the blood sugar level of patients with diabetes were higher than the normal range. However, pancreatic islet blood flow measurement is very difficult, therefore, relatively limited studies were published on the relationship between the blood circulation of the pancreas and T2DM [28]. More research is required to understand the relationship between C3, blood supply of the pancreas and T2DM in a longitudinal chronicling of C3. Preliminary studies have identified the specific statistical relationship of the radial pressure wave to cardiovascular changes during menopause and pregnancy [11, 12]. Compared to those female subjects who are not diagnosed with T2DM, female diagnosed with T2DM had significant lower C3. In this study, the decreased C3 is associated with the higher risk of T2DM. The lower C3 coincides with gestational diabetes risk during pregnancy and higher risk factor of aging [29]. Therefore, C3 may be an independent predicator of the risk of diabetes for women during pregnancy and aging.

The combination of BMI and arterial radial pressure wave is a very useful first-step screening test to identify women at risk for diabetes. Investigating if the male patients and other ethnic groups have the similar trend will be imperative in the future. In addition, due to insufficient clinical data, some risk factors associated with diabetes and blood pressure, such as Low-density lipoprotein, cholesterol, smoking, medication use, family history, and history of other cardiovascular or chronic diseases, were not considered in this study. Future research will consider these important factors at the same time to revise the predictive indicators and conduct longitudinal research. A longitudinal study of each patient may also provide additional insights into if radial pressure wave changes after diagnosis of T2DM. This may be especially beneficial in the age of telemedicine or screening in those regions with less access to medical resources [30].

In conclusion, beyond the common risk scores for type 2 diabetes to identify women at high risk, non-invasive measurement of arterial radial pressure wave provides supplementary and independent predictive value.

## Supporting information

S1 Dataset Minimal data set. (XLSX)
